# Exposure to ionizing radiation disrupts normal epigenetic aging in Japanese medaka

**DOI:** 10.18632/aging.203624

**Published:** 2021-10-13

**Authors:** Emily M. Bertucci, Marilyn W. Mason, Olin E. Rhodes, Benjamin B. Parrott

**Affiliations:** 1 Odum School of Ecology, University of Georgia, Athens, GA 30602, USA; 2 Savannah River Ecology Laboratory, University of Georgia, Aiken, SC 29802, USA

**Keywords:** epigenetic aging, DNA methylation, ionizing radiation

## Abstract

Alterations to the epigenome are a hallmark of biological aging and age-dependent patterning of the DNA methylome (“epigenetic aging”) can be modeled to produce epigenetic age predictors. Rates of epigenetic aging vary amongst individuals and correlate to the onset of age-related disease and all-cause mortality. Yet, the origins of epigenetic-to-chronological age discordance are not empirically resolved. Here, we investigate the relationship between aging, DNA methylation, and environmental exposures in Japanese medaka (*Oryzias latipes*). We find age-associated DNA methylation patterning enriched in genomic regions of low CpG density and that, similar to mammals, most age-related changes occur during early life. We construct an epigenetic clock capable of predicting chronological age with a mean error of 61.1 days (~8.4% of average lifespan). To test the role of environmental factors in driving epigenetic age variation, we exposed medaka to chronic, environmentally relevant doses of ionizing radiation. Because most organisms share an evolutionary history with ionizing radiation, we hypothesized that exposure would reveal fundamental insights into environment-by-epigenetic aging interactions. Radiation exposure disrupted epigenetic aging by accelerating and decelerating normal age-associated patterning and was most pronounced in cytosines that were moderately associated with age. These findings empirically demonstrate the role of DNA methylation in integrating environmental factors into aging trajectories.

## INTRODUCTION

Selective pressures resulting from environmental conditions modify the rate of aging and are hypothesized to contribute to variation in lifespan across species [1]. At the individual level, variation in the rate of aging reflects a decoupling of biological from chronological aging which may underlie variable timing of life history events, physiological function, and the onset of age-related disease [2, 3]. While the origins of biological-to-chronological age mismatch are unknown, evidence suggests that the environment is a key determinant [2, 4]. However, the mechanisms by which the environment contributes to variation in biological aging are not fully resolved.

One potential mechanism integrating environmental factors into variable aging trajectories is age associated patterning of the epigenome. DNA methylation represents a fundamental epigenetic modification and occurs in concert with many other epigenetic processes, including histone modifications [5]. Not only is variation in the DNA methylome linked to extrinsic factors such as exposure to contaminants [6], famine [7], season [8] and social environment [9], recent studies show compelling links between epigenetic changes and aging [2, 10, 11]. Stereotypical changes occurring with age at cytosine-guanine dinucleotides (CpGs) can be modeled to construct “epigenetic clocks” which predict chronological age with unprecedented accuracy in several vertebrate species [2, 12]. While epigenetic modifications are considered a hallmark of normal aging [13], the causal role that “epigenetic aging” might play in declines in cellular and physiological function is debated. Additional research on the basic biology of epigenetic aging in a variety of species is needed to advance a broader understanding of the underlying mechanisms driving both normal and accelerated epigenetic aging.

Certain aspects of epigenetic aging appear conserved across vertebrates, with epigenetic clocks developed in several mammals [10, 14–17], a bird [18], and most recently fish [19, 20]. Comparisons of epigenetic clocks in mice, humans, and dogs reveal functional similarities including an enrichment of age-associated loci in developmental genes [21], suggesting these clocks might converge on similar biological processes across taxa. The broad sense heritability of epigenetic age acceleration has been estimated to be up to 40% [10], although more recent evidence suggests this is an overestimation and that environmental conditions exert more influence on epigenetic age acceleration when compared to genetic components [22]. Thus, whereas phylogenetic relationships may play a role in determining the rate of epigenetic aging across species, environmental conditions are likely the most significant drivers of intraspecific variation in epigenetic aging.

Individual variation in epigenetic aging appears to be linked to numerous fundamental life history traits [12] including the precocious onset of puberty [23], menopause [24], and mortality [25]. Even more, epigenetic age appears to reflect aspects of reproductive effort, such as oocyte yield [26], number of pregnancies [27], and the birth weight of offspring [28]. Adverse environments are thought to affect plasticity in life history traits [1], and given that stressful [29] and polluted [30] environments are associated with epigenetic age-acceleration in humans, epigenetic aging presents a potential mechanism linking environmental factors and individual variation in biological aging. However, identifying causal relationships between epigenetic aging and environmental factors is fundamental to understanding the mechanistic role epigenetic processes might play in life history variation.

Alterations to the epigenome are recognized as a hallmark of aging [13, 31, 32], but whether age-associated epigenetic modifications are reflective of one or many different underlying processes is unknown. Some of the first studies reporting epigenetic change with age reported the random loss of epigenetic information over time, or epigenetic drift [33]. In contrast, epigenetic clocks rely on non-random changes to the DNA methylome over time, suggestive of a process independent of epigenetic drift [34]. Recent work demonstrates that DNA damage, namely the induction of DNA double stranded breaks (DSBs), plays an important role in the acceleration of epigenetic age [35]. Induction of DSBs in mice accelerates epigenetic aging via the relocalization of chromatin modifiers (RCM) which are recruited to aid in DSB repair [35]. During RCM, chromatin modifiers leave their original loci and do not always return with high fidelity, resulting in aberrant epigenetic patterning and the loss of epigenetic information over time [35]. Interestingly, this proposed mechanism involves the occurrence of epigenetic drift at non-random loci [35] – producing predictable patterns from random processes. This provides a potential link between the endogenous and exogenous factors which may accelerate normal aging [36]. However, additional research is needed to understand whether epigenetic aging and epigenetic drift are separate, indistinguishable, or linked processes. Further, resolving the relationship between environmental factors (i.e., those causing DSBs) is required to understand the origins of epigenetic-to-chronological age discordance and the impact it may have on variation in life history traits.

While associations between epigenetic age acceleration and disease states are well established, the proximate mechanisms regarding how epigenetic aging interacts with environmental signals is not known. To address this, we investigate the physical convergence of environmentally induced changes and age-related changes across the DNA methylome. Here, we identify signatures of epigenetic aging by sequencing DNA methylomes of a well-studied teleost, the medaka fish (*Oryzias latipes*), and compare our findings to those observed in mammals. Medaka are ideal for this type of study due to their hardiness, short lifespan, and small genome [37]. We construct an epigenetic clock *de novo* based on age-associated changes to the DNA methylome and use an outdoor replicated mesocosm experiment to analyze how chronic exposure to ionizing radiation (IR) from gamma-emitting Cesium-137 sources affects normal epigenetic aging. IR is a ubiquitous environmental stressor under which all life has evolved and is a known source of DSBs [38]. IR exposures of less than 10 mGy/day are deemed to be safe for aquatic organisms, however, lower doses are known to cause biological effects [39]. Here, we analyze how a 7-week exposure to 5, 50, and 500 mGy/day of IR alters epigenetic aging with the hypothesis that IR exposure may result in epigenetic age acceleration via the induction of RCM. Collectively, our study advances our understanding of conserved epigenetic aging patterns in vertebrates, and highlights how environmental factors, like IR, influence the aging methylome to both disrupt and accelerate normal epigenetic aging.

## RESULTS

### Characteristics of the age-associated methylome

**Figure 1:**
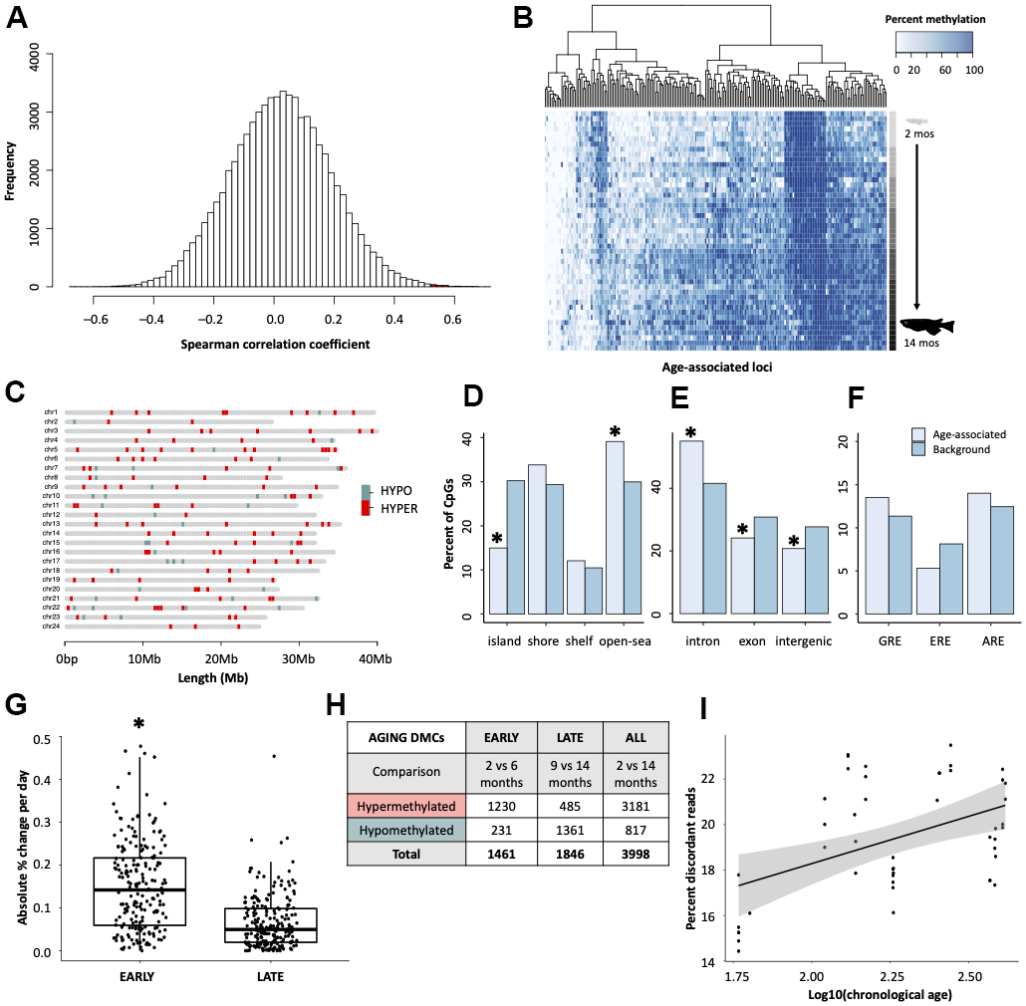
**Characterization of age-associated DNA methylation patterning in medaka hepatic tissue.** (**A**) Histogram of correlation coefficients between methylation status and age in days. Hypermethylated cytosines are shown in red and hypomethylated in blue. (**B**) Heatmap of age-associated cytosines (n = 207). Age is specified by color intensity (2-month: light gray to 14-month: black). (**C**) Distribution of age-associated cytosines across the medaka genome. Cytosines that become hypermethylated with age are shown in red and those that become hypomethylated with age in blue. (**D**–**F**) Bar plots showing comparisons between age-associated cytosines (light blue) and background (dark blue) coverage of genomic features. (**G**) Comparison of the change in methylation during early- and late-life across age-associated cytosines. (**H**) Table showing differential methylation between early- and late-life. (**I**) Differences in the percent of reads which have discordant methylation across age groups.

Spearman correlation coefficients revealed that, across the medaka methylome, 166 cytosines (0.24%) gain methylation with age (cor > 0.5) and 41 cytosines (0.06%) lose methylation with age (cor < -0.50; Figure 1A). Collectively, 0.3% of all cytosines covered were correlated with age as assessed by correlation coefficients > ± 0.5. After an FDR correction for multiple comparisons, 73 cytosines (0.11%) retained significant p-values (*p* < 0.05). Age-associated cytosines generally gain methylation with age (Figure 1B).

To determine the organization of age-associated cytosines throughout the genome, the distribution of age-associated cytosines was mapped across chromosomes. Similar to findings in other species [10, 15], age-associated changes appear to be widespread with age-associated cytosines (n = 207) present on all 24 chromosomes (Figure 1C). We then examined the genomic locations of the age-associated cytosines, as determined by Spearman correlation coefficients, with respect to their proximity to CpG islands and genic context. We observed a tendency for age-associated cytosines to fall into non-coding regions with low CpG density. For example, age-associated cytosines are enriched in CpG open-seas (*p* = 0.0049), and are significantly depleted in CpG islands (*p* = 4.6e-07) which generally harbor gene promoters [40] (Figure 1D). Relative to background, age-associated cytosines are significantly enriched in introns (*p* = 9.9e-05) and depleted in exons (*p* = 0.042; Figure 1E). However, age-associated cytosines were also slightly more likely to be in genic rather than intergenic regions (*p* = 0.024; Figure 1E). As CpGs in the human epigenetic clock are reported to co-localize with glucocorticoid response elements [29], we tested for significant deviations from the expected overlap between age-associated sites and three hormone response elements. However, enrichment of age-associated cytosines in glucocorticoid, estrogen, an androgen response elements was not detected (Figure 1F).

### Temporal dynamics of epigenetic aging

Using the top age-associated cytosines (n = 207), we determined that the mean absolute rate of change was greater in early life (2-6 months; 18.4%) when compared to later in adulthood (9-14 months; 9.5%; t = 9.65, df = 206, *p* < 2.2e-16; Figure 1G). After correcting for the number of days elapsed between each time point, medaka had an average absolute change in methylation of 0.15% per day during early life and 0.066% per day during later life, suggesting that the rate of change in methylation is more than twice as fast during early life. Additionally, the aging methylome also appears to be qualitatively different in early and later life. There are far more differentially methylated cytosines (DMCs) that become hypermethylated with age in early life (1230 DMCs) compared to later life (485 DMCs; Figure 1H). Conversely, the number cytosines which lose methylation with age is much greater in later life (1361 DMCs) relative to early life (231 DMCs; Figure 1H). Interestingly, 74.3% of cytosines which change between 2- and 14-months also become hypermethylated. Collectively, these findings demonstrate that the aging DNA methylome is qualitatively different across the lifespan and is quantitatively more dynamic during early life.

### Discordant methylation

We also examined the relationship between chronological age and discordant methylation, an indicator of epigenomic instability [41]. Genome wide levels of discordant DNA methylation were observed to increase with log-transformed age (LMM; ß = 3.01, SE = 0.66, *p* = 4.11e-05; Figure 1I), suggesting that epigenetic patterns are indeed eroded over time, consistent with epigenetic drift.

### Constructing an epigenetic clock for medaka

**Figure 2:**
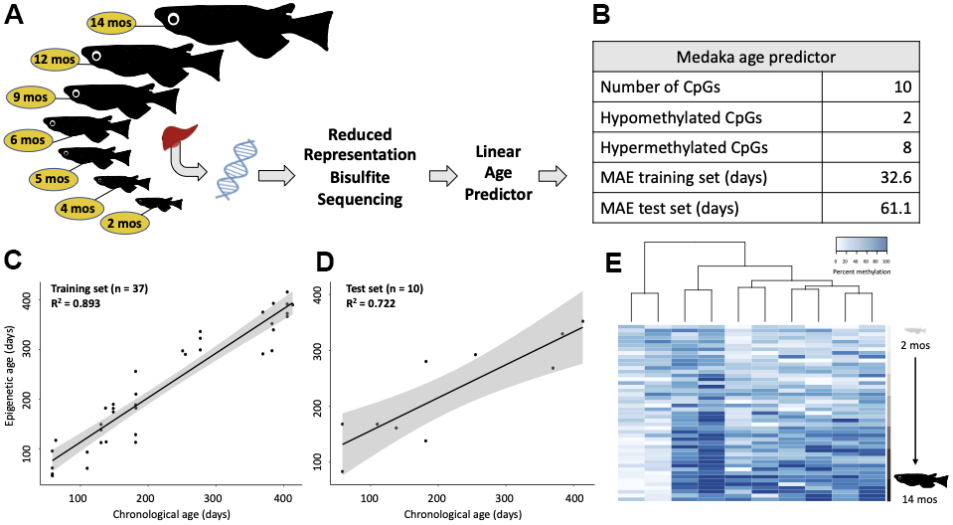
**Construction of an epigenetic age predictor in medaka.** (**A**) Conceptual diagram of the RRBS experiment. (**B**) Description and performance metrics of the medaka epigenetic clock. (**C**) Performance of the epigenetic clock on the training set (n=37) and (**D**) test set (n=10). (**E**) Heat map of the methylation of the 10 clock cytosines. Age is specified by color intensity (2-month: light gray to 14-month: black).

Training a linear model on the medaka DNA methylome revealed the highly predictable nature of age-associated remodeling (Figure 2A, 2B). We created a linear model by selecting 10 cytosines whose methylation patterns were the most highly correlated with chronological age (i.e. greatest Pearson correlation coefficients). This linear model performed well on the training set of 37 samples (R^2^ = 0.893; MAE = 32.6 days; Figure 2C) and was validated using a test set (n = 10) consisting of 1-2 samples per age group, selected at random (R^2^ = 0.722; MAE = 61.1 days; Figure 2D). When considered in the context of a 2-year lifespan, which is standard in our medaka population, this represents age-prediction accuracy within 8.4% of the lifespan. This linear model is largely (80%) comprised of cytosines which become hypermethylated with age (Figure 2E).

Additionally, we compared the performance of three different approaches to building epigenetic clocks across the training and test sets (Supplementary Figures 1–4). While the elastic net approach produced a model which was effective at predicting age in the test set (R^2^ = 0.76, MAE = 60.9 days) it was also very overfit to the training set (R^2^ = 0.99, MAE = 3.9 days; Supplementary Figures 1, 2 and Supplementary Table 1). Conversely, the PCA based epigenetic clock was a relatively weak predictor of chronological age in both the training (R^2^ = 0.77, MAE = 48.7 days) and test sets (R^2^ = 0.64, MAE = 73.9 days; Supplementary Figures 3, 4 and Supplementary Table 1). The approach that minimized both overfit and mean absolute error (MAE) of the training set was determined to be a linear model (Supplementary Table 1).

### Environmentally accelerated epigenetic aging

**Figure 3:**
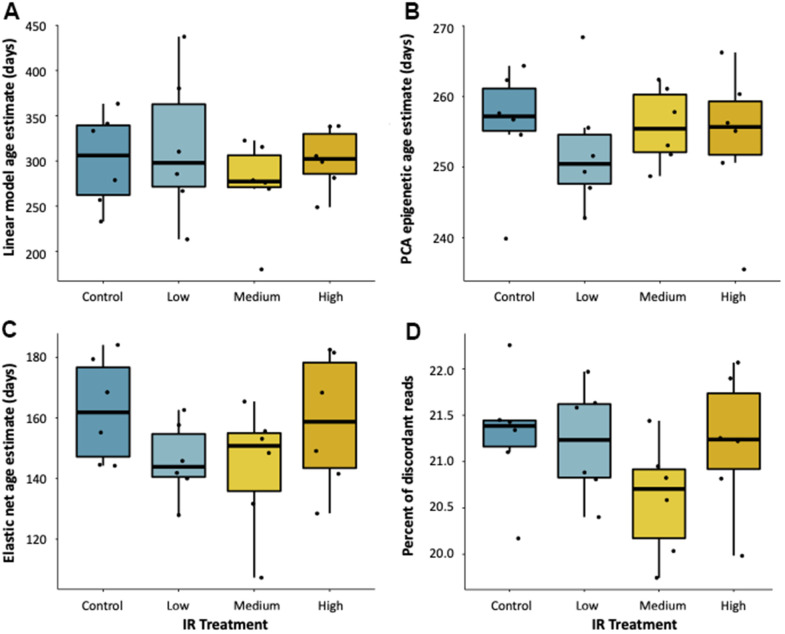
**Effect of ionizing radiation on age-associated DNA methylation.** Epigenetic age estimates for fish exposed to 7-weeks of ionizing radiation at various dose rates (0, 5, 50, and 500 mGy/day) as predicted by (**A**) a linear model, (**B**) PCA, and (**C**) elastic net age predictors. (**D**) Percent of reads with discordant methylation across exposure groups.

To test if exposure to an environmental stressor accelerates epigenetic aging, we applied all three of our epigenetic clocks to fish exposed to ionizing radiation for seven weeks at 5, 50, and 500 mGy/day in a Low Dose Irradiation Facility (LoDIF). There was no difference in age prediction in fish exposed to IR relative to unexposed controls (Figure 3A–3C). Additionally, IR exposure did not affect the percentage of reads with discordant methylation (Figure 3D), nor did it affect survival rate across treatments.

### Environmental effects on the aging methylome

**Figure 4:**
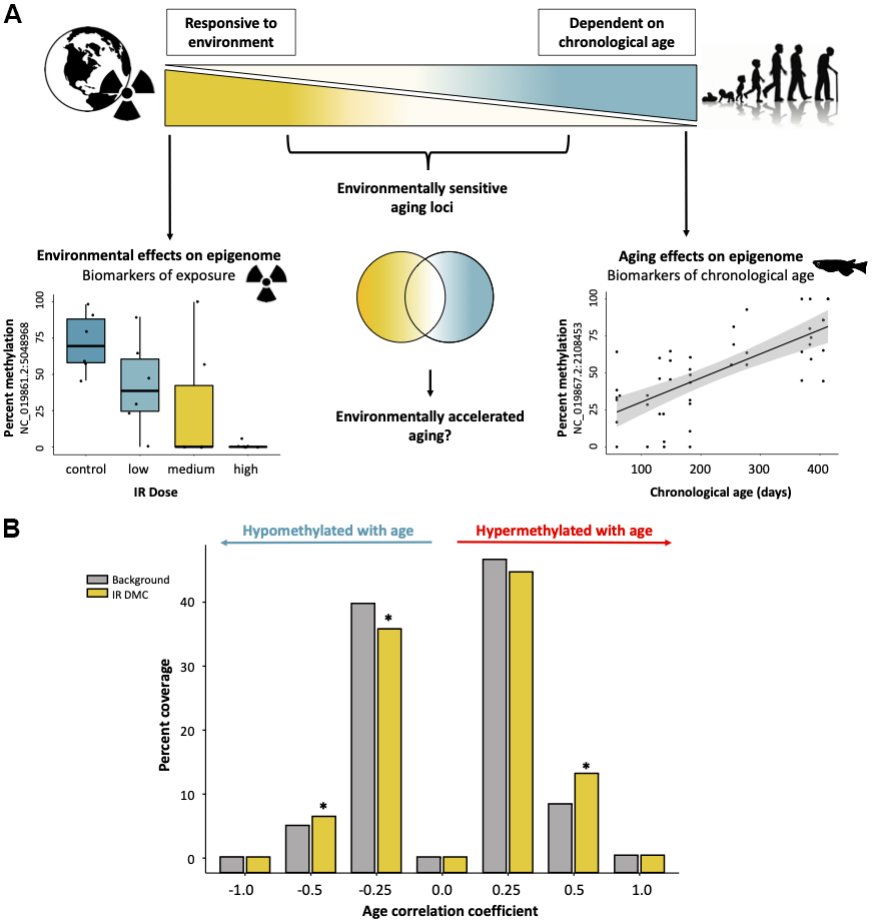
**Interactive effects of exposure to ionizing radiation and age on the medaka DNA methylome.** (**A**) Diagram of a hypothetical continuum of cytosines with cytosines affected strongly by age on one end and those which are highly environmentally responsive on the other. (**B**) Distributions of cytosines affected by IR exposure along the continuum of association with chronological age. Background represents the number of overlapping CpGs in the two datasets (age and radiation exposure).

We hypothesized that the influence of age and environmental factors on cytosine methylation might exist along an opposing continuum with strongly age-associated cytosines independent of environmental influences on one end and cytosines affected by the environment and less likely to be affected by age on the other. Along this continuum, we posited that a subset of cytosines exist that are affected both by age and environmental exposures (Figure 4A). To test this hypothesis, we first sorted cytosines into bins according to the degree to which their methylation status was correlated with age to form an aging continuum. We then determined which cytosines become differentially methylated after exposure to ionizing radiation and identified 8,595 cytosines whose methylation status is significantly affected by exposure to at least one dose of IR (5, 50, and 500 mGy/day) relative to our background exposure control. Between the aging and LoDIF datasets there were 58,825 cytosines (85.2%) which were dually represented. Of the IR DMCs, 1072 (12.5% of IR DMCs) were also represented in our aging dataset (out of a total of 69,064 cytosines). We then tested if cytosines which were differentially methylated after IR exposure were enriched in age-associated bins relative to background representation. Consistent with our hypothesis, we found that cytosines which become differentially methylated due to IR exposure were overrepresented in bins harboring cytosines with weak age-associations, ranging from –0.25 to –0.5 (*p* = 0.035) and 0.25 to 0.5 (*p* = 8.9e-08; Figure 4B). Interestingly, we also detect a depletion of IR DMCs in cytosines which have correlation coefficients between 0.0 and –0.25 (*p* = 0.0045; Figure 4B).

**Figure 5:**
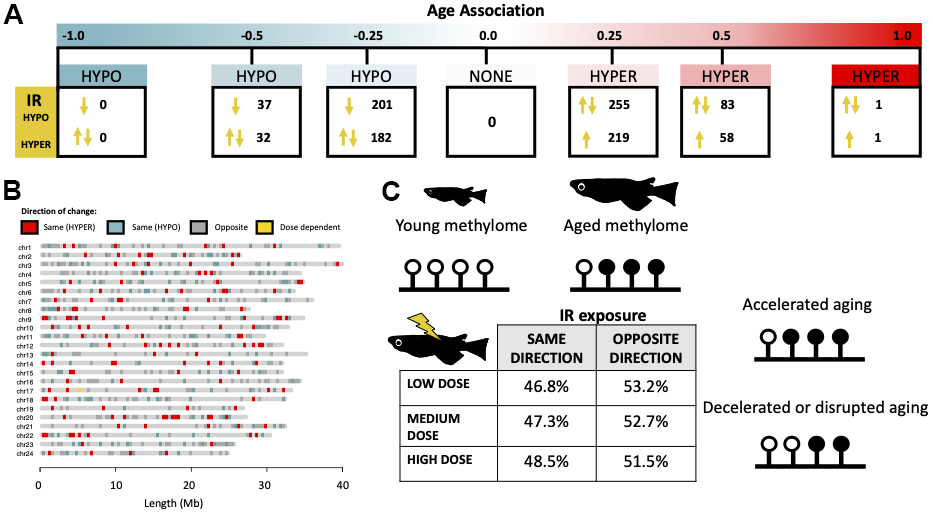
**Directionality of IR-induced changes to methylation status in the context of normal epigenetic aging.** (**A**) Distribution of cytosines which become differentially methylated from IR exposure along the continuum of association with chronological age. Arrows signify whether the IR induced change is in the same or opposite direction as changes induced by age. (**B**) Genomic distribution of cytosines which become differentially methylated with IR exposure in the same (red/blue) or opposite (gray) direction as age-related changes. Cytosines which become hypermethylated with both age and IR exposure are shown in red and those which become hypomethylated in blue. Cytosines with direction dependent on IR dose are shown in yellow. (**C**) Table showing the percent of cytosines whose methylation changes in the same and opposite direction across dose rates and a conceptual diagram of the hypothesized effect this could have on the aging methylome.

We further investigated the link between exposure to IR and epigenetic aging by assessing the directionality of IR-induced methylation in relation to changes occurring during normal aging. Of the cytosines which were both affected by IR and age (Figure 5A), we found that IR exposure resulted in methylation shifts reflective of both accelerated and decelerated epigenetic age (Figure 5A) and these effects are observed across the genome (Figure 5B). Interestingly, there is an increased incidence of IR exposure resulting in deceleration of normal epigenetic aging in cytosines with weak positive age-associations, ranging from zero to 0.25 (*p* = 1.3e-05) and 0.25 to 0.5 (*p* = 0.043; Figure 5A). There were no dose dependent effects on the direction of change (Figure 5C).

## DISCUSSION

Here, we characterize age associated patterning of the medaka DNA methylome and model these patterns to construct an epigenetic clock that predicts chronological age with an accuracy of 8.4% of the lifespan. We demonstrate that epigenetic aging in medaka shares similarities with other species such as mice and humans [12]. Most notably, age associated cytosines tend to be located in regions with lower CpG density and in non-coding regions, similar to findings in mice [15]. However, the opposite trend is seen in dogs [21], which may in part be a product of targeting conserved mammalian sequences rather than RRBS. In contrast to humans, we do not observe an enrichment of age-associated methylation patterning in glucocorticoid receptor response elements, nor in the response elements of other nuclear hormone receptors, suggesting fundamental differences in aging programs may also be present [29]. How these similarities and differences translate into functional variation across aging and life history trajectories warrants further investigation.

Epigenetic aging in medaka occurs approximately twice as fast early in life when compared to rates in mature fish. This is consistent with findings in mice, humans, and dogs suggesting that the methylome is most dynamic early in life and corresponds to physiological processes (i.e. growth, maturation) in similar ways across species [21]. In addition to increased rates of change at specific cytosines, we find that a greater number of cytosines incur gains of methylation during early life. These findings raise the potential that epigenetic aging occurs on a qualitative scale in which different cytosines acquire or lose age-associated methylation patterning semi-independently of aging rate. Our data and those of others [2, 42] are consistent with studies demonstrating that biological aging, as measured by telomere length, is also more dynamic during early life [43–47]. In most cases, increased rates of telomere attrition are associated with faster growth early in life and are thought to reflect trade-offs between growth and longevity [48–50]. Whereas positive correlations between accelerated epigenetic aging and individual variation in body size have been reported in humans [51], these linkages have not been explored in the context of variable life history strategies present in other ecological systems [12]. Given that evolutionary theories of aging, such as antagonistic pleiotropy, emphasize the importance of maximizing fitness during early life [52, 53], understanding the role of the environment in determining rates of epigenetic aging at different life stages stands to better inform our understanding of the mechanisms driving adaptive plasticity in life histories. Studies examining how epigenetic aging trajectories, both in a quantitative (e.g., rate) and qualitative (e.g., variable subsets of cytosines acquiring age-associated patterns) sense, are affected in early life are clearly needed to advance our understanding regarding both the origins of epigenetic-to-chronological age discordance and the potential adaptive roles it might play in nature.

Double stranded DNA breaks have recently been shown to accelerate epigenetic aging in mice [35], and based on these findings, we hypothesized that chronic IR exposure, a known source of DSBs [54], might also result in accelerated aging. Previous research in humans has demonstrated a relationship between radiation treatment in breast cancer patients and increased epigenetic age [55]. However, after a 7-week exposure to three doses of IR, we do not observe any accelerated epigenetic aging in medaka. However, it is also possible the negative result reflects a weakly trained epigenetic clock or is a constraint of our low sample size. Further, given that the rate of epigenetic aging slows with chronological age [2], it is also possible that the timing of IR exposure dictates the impact of on epigenetic aging trajectories. The fish in this study were adults, thus exposure during earlier windows of development could have been more influential. Experiments assessing different exposure durations at different life stages are needed to determine if environmentally relevant sources of DSBs result in epigenetic-to-chronological age discordance.

Although the epigenetic clock itself was not affected, IR exposure did interact with the aging DNA methylome. Cytosines with weak associations with age were more likely to be affected by IR exposure. This result supported our hypothesis that cytosines strongly affected by age would be relatively insensitive to environmental factors. However, contrary to our hypothesis, we find that the majority of cytosines which become weakly hypermethylated with age respond to IR exposure in the opposite direction of normal epigenetic aging. IR has long been considered a general accelerant of normal aging [56], and yet our findings suggest the effects of IR on the aging epigenome are more nuanced than previously realized and may both accelerate and decelerate normal patterns of aging at specific cytosines across the DNA methylome. The response of epigenetic aging trajectories to ecologically (and evolutionarily) relevant exposures will likely inform a broader understanding of the potentially adaptive role of epigenetic age acceleration in nature.

The current study demonstrates that specific aspects of epigenetic aging are broadly conserved across vertebrates, while other aspects appear more divergent. Given the similarities between the medaka epigenetic clock with those developed in mammals, epigenetic aging is likely to have a conserved functional role in the aging process, although more work examining the causal roles of epigenetic aging is needed. Further, using these predictable patterns we have developed a medaka epigenetic clock which can be used to predict chronological age as well as measure biological age acceleration after exposure to environmental stressors. We report a strong dependence of epigenetic aging patterns on early life changes in the methylome, suggesting that the timing of exposure may determine the sensitivity to environmental stressors. Exposure to IR at an earlier time point may have had greater impacts on epigenetic aging. However, the relatively small sample sizes for both the epigenetic clock (n = 47) and IR exposure experiments (n = 24) prevent us from making broad conclusions about the effects of IR on epigenetic aging. Overall, the findings in this study demonstrates the nuanced relationship between the environment and biological aging and establishes a model by which environment-by-aging interactions can be further explored.

## MATERIALS AND METHODS

### Development of the epigenetic clock

### Animal husbandry and rearing

All procedures involving fish husbandry and animal experiments were approved by the University of Georgia’s IACUC (protocol A2018 09-007-Y3-A2). Medaka were bred under optimal conditions (24° C, 16L:8D) to produce offspring which were raised under similar conditions. For the construction of the epigenetic clock, male fish aged 2-months (n = 7), 4-months (n = 6), 5-months (n = 6), 6-months (n = 8), 9-months (n = 6), 12-months (n = 8), and 14-months (n = 6) were sacrificed using an overdose of sodium bicarbonate buffered Tricaine (MS-222 300 mg/L). Fish were immediately necropsied, and hepatic tissue stored in RNAlater at -80° C or -20° C. The sex of fish was determined using sexually dimorphic fin structure and gross morphology of gonads. Animals at 2-months of age had not yet reached sexual maturity so PCR amplification of the Y-chromosome specific gene, DMY, was used to determine genetic sex [57].

### DNA extraction

DNA was extracted using a modified column protocol. Whole livers were homogenized in a lysis buffer (4M guanidinium thiocyanate, 0.01M Tris-hydrochloric acid (pH 7.5), and 2% beta-mercaptoethanol) using a Mini-Beadbeater (BioSpec, Bartlesville, OK) and stainless steel beads for 2 minutes at 30 Hz in. Resulting lysates were centrifuged, and supernatants were transferred to spin columns containing a fiberglass filter (Epoch Life Science, Missouri City, TX). Column-bound DNA was washed twice with wash buffer (60% ethanol, potassium acetate (162.8 mM)), and tris-hydrochloric acid (27.1 mM, pH 7.5), diluted with 60% (v/v) of ethanol) and eluted in 40 μl of TE buffer. DNA concentrations were quantified using a Qubit fluorometer 2.0 (Invitrogen, Carlsbad, CA) and purity was assessed on a Nanodrop spectrometer (Thermo-Scientific, Waltham, MA) using absorbance ratios at 260/280 and 260/230. DNA was stored at -20° C until library preparation.

### Reduced representation bisulfite sequencing

Reduced representation bisulfite sequencing (RRBS) was used to analyze the DNA methylome, which is preferred over whole genome bisulfite sequencing due to the enrichment of areas with high CpG content [58]. The RRBS libraries were prepared using the Diagenode Premium RRBS Kit (#C02030032, Diagenode, Denville, NJ) with the following exceptions: 200 ng of genomic DNA was used for input and the equation cycle threshold (Ct) + 2 was used to determine the number of PCR cycles needed for final library amplification. AMPure XP Beads (Beckman Coulter, Brea, CA) were used for all steps in the Diagenode protocol requiring size selection or clean-up of products. Final libraries were stored at -80° C until sequencing. Libraries were prepared in two separate batches, 2-, 6-, 12-months and 4-, 5-, 9-, 14-months, respectively.

### Sequencing, quality control and bisulfite conversion efficiency

Libraries were submitted to the Georgia Genomics and Bioinformatics Core where they were further assessed for quality and concentration using a Fragment Analyzer (Agilent, Santa Clara, CA). Libraries were pooled and sequenced single-end for 75 cycles on one high-output flow cell of an Illumina NextSeq 500 with 5% PhiX control (Illumina) for the first batch and sequenced single-end for 100 cycles on one high-output flow cell of the Illumina NextSeq 2000 with 20% PhiX control added for the second batch for greater base pair diversity. The sequencing run for the first batch generated 225 million reads across samples, with 92% of reads falling above the high-quality threshold of Phred score >30. The second batch generated 525 million reads across samples with 91% at or above the high-quality threshold. Read quality was assessed using FastQC (v0.11.5). Adapters and low-quality sequences (Phred score <25) were trimmed using TrimGalore! (v 0.4.5; --rrbs) and 4bp at the 3’ end was removed due to low overall quality scores of the 3’ end of reads. The efficiency of the bisulfite conversion was determined to be >98% using MethylKit’s (v1.10.0) [59] conversion statistics in Program R (v3.5.1). Data from the prepared RRBS libraries were made publicly available using NCBI’s Sequence Read Archive (BioProject: PRJNA716946) [60].

### Alignment and Methylkit

Using Bismark (v0.20.0) [61] the medaka reference genome (ASM223467v1) was indexed for bisulfite conversion, and reads were aligned to this indexed reference using Bismark with an overall alignment rate of 50-68%. Using the Bismark methylation extractor, BAM files containing methylation calls for each sample were produced, sorted using SAMtools (v 0.1.19) [62], and used as input for Bioconductor’s (v 3.9) package MethylKit (v3.12) [59] in Program R (v3.5.1). In MethylKit, we first filtered out cytosines which were covered at a depth of less than 5x or greater than 100x and normalized samples by coverage using MethylKit’s normalizeCoverage function to prevent bias arising from PCR or over-sampling of specific individuals, respectively. Cytosines from opposite strands were not merged together (option destrand = FALSE). For characterization of the aging methylome, cytosines which were covered at a depth of at least five reads in at least five samples per age group (i.e. at least 74.5% of samples) were selected resulting in a subset of 90,566 cytosines. Missing data was imputed using k-nearest neighbor imputation (k = 4) by utilizing the function ‘impute.knn’ from R package impute [63]. Invariable cytosines were removed using the ‘nearZeroVar’ function in the package caret [64], resulting in 69,064 cytosines which were used for further analysis.

### Correlation between age and methylation status

We used Program R (v3.5.1) and the function ‘corr.test’ from package psych (v1.8.12) to run independent Spearman and Pearson correlations for each cytosine which passed filtering. Correlation coefficients between the percent methylation and age in days were computed and significance values were calculated using a false discovery rate (FDR) correction for multiple comparisons. Spearman correlations were used to determine the age-associated methylome in order to describe both linear and nonlinear relationships with age. Pearson correlations were used to determine inclusion of cytosines in a linear age predicting model. Cytosines with a correlation coefficient > 0.5 or < -0.5 were considered age-associated.

### Determination of differentially methylated cytosines

To determine differentially methylated cytosines we used MethylKit’s (v3.12) ‘calculateDiffMeth’ function. Differentially methylated cytosines were those which had at least 25% difference in methylation between age groups with a q-value less than or equal to 0.01. Three individual comparisons were made between age groups (2-month vs. 6-month, 9**-**month vs. 14-month, and 2-month vs. 14-month).

### Characterization of genomic location

Genomic locations for age-associated cytosines determined by Spearman correlations were characterized using Galaxy’s coverage function (Galaxy Version 2.29.0) [65]. To determine the genic context of each cytosine, we used the medaka reference genome annotation (ASM223467v1) to assign cytosines as lying in introns, exons, or intergenic (not in exon or intron) regions. The genomic coordinates were compared against the age-associated cytosines and background cytosines using the coverage function in Galaxy (version 20.05) [65]. Similarly, CpG island (CGI) context was determined by first producing a BED file with CGI coordinates using Galaxy’s function cpgplot (Galaxy Version 5.0.0) [65] according to a sliding window of 100 bp (minimum size of CGI = 200 bp, Minimum average observed to expected ratio = 0.6, Minimum average percentage of G plus C = 50.0). Using bedtools ClosestBed (Galaxy Version 2.29.0) [65], we were then able to assign each cytosine to either a CpG island (within CGI), CpG shore (area of 2kb flanking CGI), CpG shelf (area from 2kb-4kb flanking CGI), or as an open-sea CpG (further than 4kb to nearest CGI). Enrichment above background was performed with binomial tests using the full dataset of 69,064 covered cytosines as the background.

### Hormone response elements (HRE)

To determine if age-associated cytosines were enriched in hormone response elements (HREs), we first established the coordinates of prospective HREs in the medaka genome using PoSSuM search (Version 2.0). Raw PFM files were downloaded from JASPAR [66] for the estrogen (ERE; MAO112.2), glucocorticoid (GRE; MAO113.3), and androgen response elements (ARE; MA0007.2). The raw PFM files were transformed into PPM files (divide the frequency by the total count for that position) and then into PSSM files (Log2(PPM value/0.25)) where the PPM value is the count/total and the 0.25 is the null expected frequency of that base. These PSSM files were input into PoSSum search (-pr -format tabs -pval 0.0001 -lazy -uniform -lahead -dna) and compared against the medaka reference genome to identify prospective HREs with a p-value < 0.0001 considered a significant HRE. The genomic coordinates from the prospective HRE sites were extracted into a BED file and compared against the age-associated cytosines and background cytosines using the coverage function in Galaxy (version 20.05) [65] to determine coverage in a 2kb window surrounding each cytosine (1kb upstream, 1kb downstream). Age-associated cytosines were considered to be overlapping with an HRE if any bases within the 2kb window overlapped with a given HRE. Enrichment above background was performed using binomial tests using the full dataset of 69,064 covered cytosines as the background.

### Early vs late rate of change

Using the cytosines with the greatest Spearman correlations with age (cor > 0.5 or < -0.5; n = 207), we calculated the average absolute rate of change in methylation (absolute[% methylation late - % methylation early]) between each of the age groups (2- to 6-months, 9- to 14-months). Due to the differences in the amount of time elapsed between early (2- to 6-months) and late (9- to 14-months) time points, we normalized the average absolute rate of change by number of days between each age interval. This ‘daily’ average absolute rate of change was compared between the early and late time points using a paired t-test.

### Developing an epigenetic age predictor

To build the epigenetic clock, we built three different epigenetic age predictors using the same subset of cytosines (n = 69,064) and identical training and test sets. Models were compared based on their MAE and R^2^ in training and test sets (Supplementary Table 1). Overfit was assessed by comparing MAE of the training and test set using a t-test (Supplementary Table 1).

First, we built a linear model using the lm function in the stats package (v3.6.2) in Program R (v3.5.1). Variables used in the model were selected based off of the strength of Pearson correlation coefficients determined by individual cytosines relationship with un-transformed age within the training set (n= 37) calculated using the function corr.test from package psych (v1.8.12). Cytosines within 100 bp from another cytosine already included in the model were removed and replaced with the cytosine with the next highest correlation coefficient. The top 10 cytosines were chosen as this value minimized mean absolute error in the test set (n = 10).

Second, we used the GLMNET package (v1.9-9) [67] in Program R (v3.5.1). This approach is similar to those used for epigenetic clocks developed in human [10] and mouse models [15]. We used an elastic net penalized regression model (alpha = 0.5, family = gaussian) to select cytosines and assign penalties to individual model coefficients. We used a leave-one-out (nfolds = nrow) cross validation to select the optimal lambda value (value resulting in minimum mean error) for the model on the training set (n = 37 samples). The model was verified on a test set (n = 10; 1-2 sample from each age group) that was selected at random. Age (in days) was log10 transformed for this approach.

Lastly, we created an age predictor based off of a principal components analysis (PCA) of age-associated cytosines in the training set (as determined by Spearman correlation coefficients > 0.5 or < -0.5; n = 304), similar to the approach done in Lu et al. 2020 [68]. The first principal component (PC1) explained 30.01% of the variation and was used as a metric for age. We defined the linear relationship between PC1 and chronological age in the training set (n = 37) as the epigenetic age predictor. The predictive model was validated on the test set (n = 10).

### Testing the epigenetic clock

### Age prediction in fish exposed to ionizing radiation

Six month old male fish were exposed to low doses of ionizing radiation for 7 weeks at the Savannah River Ecology Laboratory’s Low Dose Irradiation Facility (LoDIF) [39]. The irradiation protocol is outlined in Bertucci et al. (2020) with slight modifications [69]. Briefly, eggs were collected during a 14-day window in January and February 2019 and reared in mixed-sex cohorts as described above. Males were separated and put into 19 L containers (15 fish/container) and placed under irradiators equipped with Cesium-137 sources emitting radiation at approximately 5, 50 and 500 mGy/day [69]. During the exposure period, fish were fed three times per week with Tetramin and were given a fresh flow of water for an hour at each feeding. Radiation sources were turned off briefly during feeding. After a 7-week exposure, fish were immediately euthanized with an overdose (300 mg/L) of Tricaine/MS-222 and immediately necropsied. DNA from hepatic tissue (n = 24) was isolated and library preparation for RRBS was performed as described above. Sequencing was performed at the GGBC single-end for 75 cycles on one high-output flow cell of an Illumina NextSeq 500 with the addition of 20% Illumina PhiX control spiked in. Data from the LoDIF RRBS libraries were made publicly available using NCBI’s Sequence Read Archive (BioProject: PRJNA717041) [70]. Reads were filtered and aligned as described above. Sequencing resulted in 590,644 cytosines which were covered at a depth of 5x-100x reads in at least 5 samples per treatment group and 1,510,504 cytosines which were covered at a depth of 5x-100x reads in at least one sample. Missing data was imputed as described above using KNN imputation (k = 5). We used all three epigenetic age predictors to determine epigenetic age of the 24 samples from the LoDIF experiment. Any cytosine missing from the dataset across all samples was left as a zero value in the epigenetic age predictor models. Epigenetic age predictions were compared between groups using analysis of variance tests (ANOVA).

### Determination of IR differentially methylated cytosines

MethylKit’s (v1.10.0) ‘calculateDiffMeth’ function was used to determine differentially methylated cytosines between control and exposure groups using the 590,644 cytosines covered across at least five samples in each treatment group. Differentially methylated cytosines (DMCs) were those which had at least 25% difference in methylation between exposure groups with a q-value less than or equal to 0.01. Three individual comparisons were made between control and treatment groups: Control vs Low (5 myGy/day), Control vs Medium (50 mGy/day), and Control vs High (500 mGy/day).

### Overlap of age-associated sites with IR DMCs

We determined the overlap between the age-associated cytosines (as determined by Spearman correlations) and the IR DMCs and tested for enrichment in age-associated bins (correlation coefficients –1.0 to –0.5, –0.5 to –0.25, –0.25 to zero, zero, zero to 0.25, 0.25 to 0.5, and 0.5 to 1.0) using binomial tests. Enrichment above background was done by comparing the overlap of the LoDIF dataset to the aging dataset of 69,064 covered cytosines as the background.

### Percentage of discordant reads

The percentage of discordant reads (PDR) was calculated using the ‘methylation_consistency’ extension in Bismark (v0.20.0, Babraham Bioinformatics). The number of reads containing at least 2 CpGs which had concordant (0-9% or 91-100% methylated) or discordant (10-90% methylated) patterns of DNA methylation was determined and PDR was calculated as the number of discordant reads divided by the total number of reads containing at least 2 CpGs. A linear mixed effects model (LMM) was used to evaluate the relationship between PDR and log-transformed age including a random effected of library batch. The LMM was run in the lme4 package and p-value extracted using the package lmerTest [71].

### Data availability

Data used to support the claims in this manuscript are publicly available in the NCBI Sequence Read Archive (SRA) under BioProjects PRJNA716946 and PRJNA717041.

### Ethics approval

All procedures involving fish husbandry and animal experiments were approved by the University of Georgia’s IACUC (protocol A2018 09-007-Y3-A2).

### Disclaimer

This report was prepared as an account of work sponsored by an agency of the United States Government. Neither the United States Government nor any agency thereof, nor any of their employees, makes any warranty, express or implied, or assumes any legal liability or responsibility for the accuracy, completeness, or usefulness of any information, apparatus, product, or process disclosed, or represents that its use would not infringe privately owned rights. Reference herein to any specific commercial product, process, or service by trade name, trademark, manufacturer, or otherwise does not necessarily constitute or imply its endorsement, recommendation, or favoring by the United States Government or any agency thereof. The views and opinions of authors expressed herein do not necessarily state or reflect those of the United States Government or any agency thereof.

## Supplementary Material

Supplementary Figures

Supplementary Table 1
