# Supplementary material for: Exposure to ionizing radiation disrupts normal epigenetic aging in Japanese medaka
Source: Aging (Albany NY). 2021 Oct 13;13(19):22752–71. doi: 10.18632/aging.203624 (PMC8544305; doi:10.18632/aging.203624)
Supplement: Supplementary Figures [file aging-13-203624-s001.pdf]

SUPPLEMENTARY FIGURES

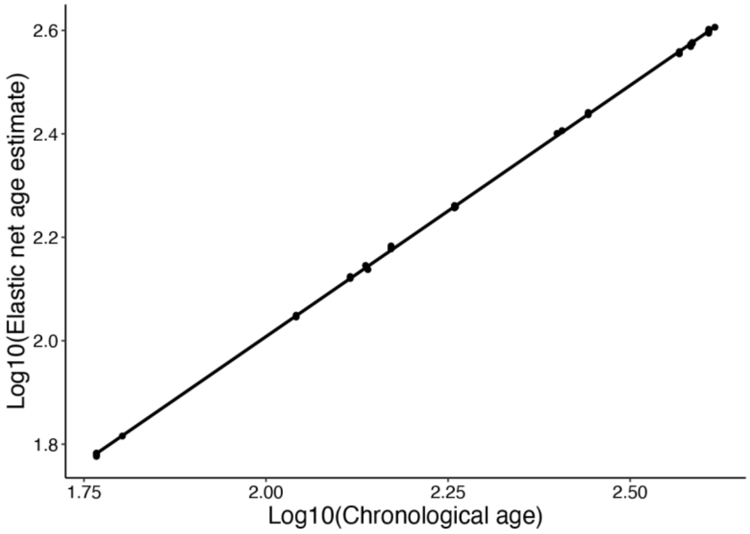

Supplementary Figure 1. Performance of the elastic net based epigenetic age predictor on the training set (n=37). R^2=0.99, MAE=3.9 days.

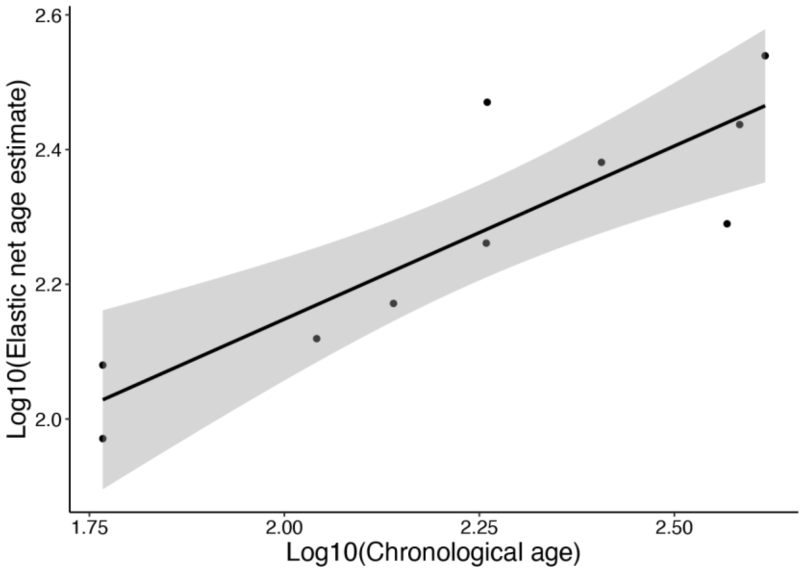

Supplementary Figure 2. Performance of the elastic net based epigenetic age predictor on the test set (n = 10). R^2 = 0.76, MAE = 60.9 days.

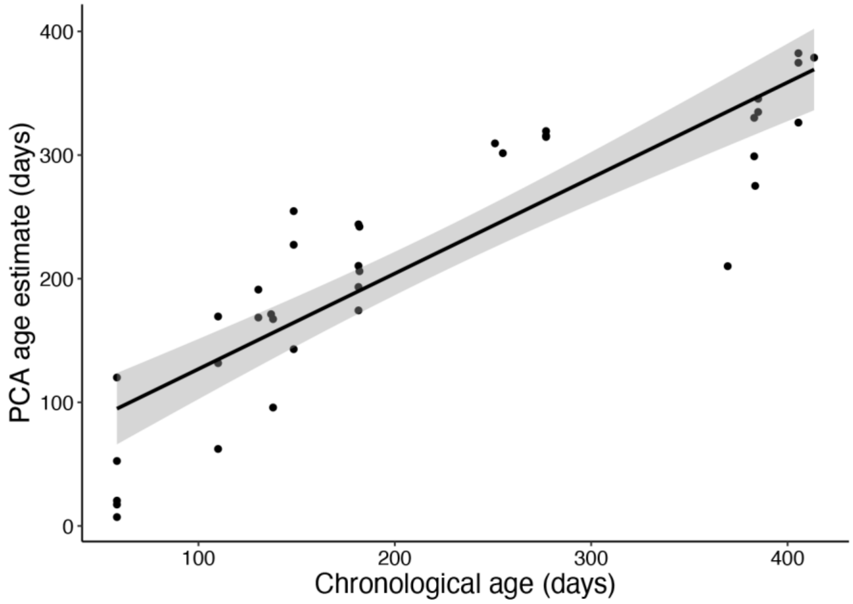

Supplementary Figure 3. Performance of the PCA based epigenetic age predictor on the training set (n = 37). R^2 = 0.77, MAE = 48.7 days.

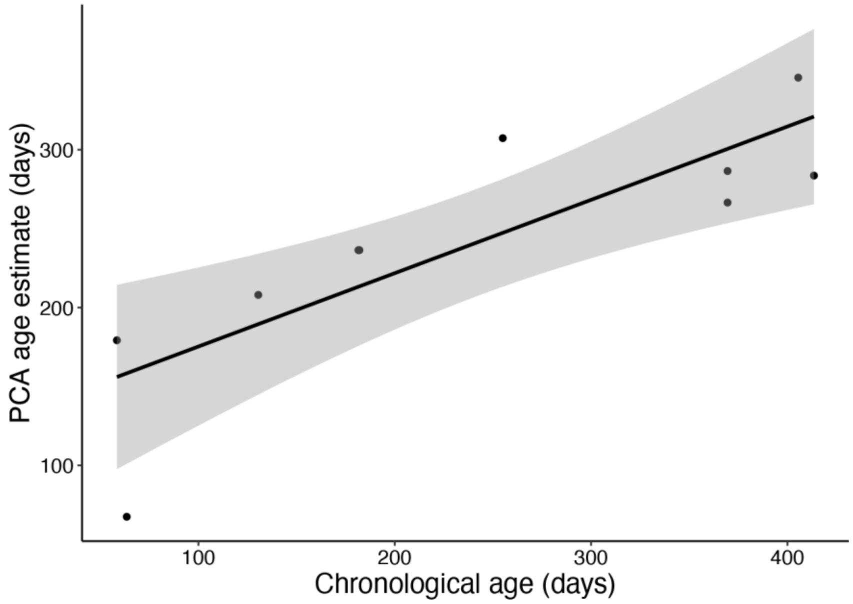

Supplementary Figure 4. Performance of the PCA based epigenetic age predictor on the test set (n = 10). R^2 = 0.64, MAE = 73.9 days.
