# Supplementary material for: Exposure to ionizing radiation disrupts normal epigenetic aging in Japanese medaka
Source: Aging (Albany NY). 2021 Oct 13;13(19):22752–71. doi: 10.18632/aging.203624 (PMC8544305; doi:10.18632/aging.203624)
Supplement: Supplementary Table 1 [file aging-13-203624-s002.pdf]

SUPPLEMENTARY TABLE

Supplementary Table 1. Comparison of overfit of epigenetic clock building approaches.

Clock building approach	Number of CpGs included	Training set MAE	Training set R^2	Test set MAE	Test set R^2	Overfit? (t-test p-value)
Elastic net	52	3.9	0.99	60.9	0.76	Yes (0.011)
Linear model	10	32.6	0.89	61.1	0.72	Yes (0.021)
PCA	304	48.7	0.77	73.9	0.64	No (0.072)
